# The Complexities of Diagnosis with Co-Existing Gaucher Disease and Hemato-Oncology—A Case Report and Review of the Literature

**DOI:** 10.3390/jcm12175518

**Published:** 2023-08-25

**Authors:** Paulina Sudul, Beata Piatkowska-Jakubas, Lukasz Pawlinski, Krystyna Galazka, Tomasz Sacha, Beata Kiec-Wilk

**Affiliations:** 1 University Hospital, 30-688 Krakow, Poland; 2 Unit of Rare Metabolic Diseases, Department of Metabolic Diseases, Jagiellonian University Medical College, 30-688 Krakow, Poland; 3 Department of Hematology, Jagiellonian University Medical College, 30-501 Krakow, Poland; 4 European Reference Network for Rare Metabolic Disease MetabERN, 30-688 Krakow, Poland; 5 Department of Pathomorphology, Jagiellonian University Medical College, 31-531 Krakow, Poland

**Keywords:** genetics, biomarkers, molecular diagnosis, coordination of care, adult-oriented medical care, enzyme replacement therapy, Gaucher disease, multiple myeloma

## Abstract

Hematological abnormalities are the most common early symptoms of Gaucher disease (GD), with an increased risk of hematopoietic system malignancies reported in patients with GD. GD may be associated with monoclonal and polyclonal gammopathies; however, the mechanism of association of GD with multiple myeloma (MM) remains uncertain. Enzyme replacement therapy (ERT) has been shown to improve patients’ cytopenia and it seems to facilitate anti-myeloma therapy in patients with co-occurring GD and MM. Although it is necessary to demonstrate the deficiency of enzymatic activity, as well as using genetic tests to finally diagnose GD, due to changes in the blood count image, bone marrow biopsy is still a frequent element of the GD diagnosis procedure. The diagnosis of GD is often delayed, mainly due to the heterogeneity of the histopathological picture of bone marrow biopsy or overlapping hematological abnormalities. Unrecognized and untreated GD worsens the response of a patient with an oncological disease to targeted treatment. We present a literature review, inspired by the case of a Caucasian patient initially diagnosed with MM and later confirmed with comorbid GD type 1 (GD1). We would like to point out the problem of underdiagnosis and delay in patients with GD.

## 1. Introduction

Gaucher disease (GD) is a hereditary lysosomal storage disease, inherited in an autosomal, recessive manner. A deficiency in the activity of the lysosomal enzyme leads to glucosylceramide accumulation in tissue macrophages, causing the development of chronic inflammation [1]. It is commonly associated with the enlargement of internal organs and bone complications. However, it should be emphasized that the most common early symptoms are related to hematological abnormalities, such as abnormal blood counts and gammopathy development [2]. The presentation of GD symptoms is very heterogeneous, with its forms ranging from asymptomatic to fatal. The most common variation of GD is non-neuronopathic type 1, causing the symptoms in many systems without the primary involvement in the nervous system [1,3].

Some publications prove that diseases associated with the accumulation of metabolic products predispose one to the occurrence of certain cancer types [4]. In GD, there is an accumulation of bioactive glycosphingolipids, which are considered carcinogenic [5]. However, there are several hypotheses about the potential carcinogenesis-inducing mechanisms in GD patients, such as the chronic stimulation of the immune response by macrophages, chronic inflammatory process, increased free radical production, autophagy, and mitochondrial dysfunction [6,7,8,9]. The elevated levels of IgG and IgM antibodies observed here suggest basal lymphoproliferation [10]. Effective diagnostics and the ability to quickly implement treatment have significantly extended the life expectancy of patients with GD, and with it, the risk of developing cancer throughout their lives [11].

The increased risk of several neoplasms, especially hematological malignancies like multiple myeloma (MM), in Gaucher patients, was reported previously [12,13]. MM is a neoplasm characterized by the proliferation of malignant plasma cells producing abnormal antibody monoclonal proteins, called M proteins [14]. The accumulation of M proteins in the lysosomes of various cells, such as histiocytes in the bone marrow or other tissues, can lead to the development of plasma cell dyscrasia and other lymphoproliferative disorder called crystal storage histiocytosis (CSH) [15]. The differential diagnosis of this condition, apart from excluding hematological causes, should include lysosomal storage disorders, because these histiocytes can mimic Gaucher cells and that is why they are sometimes named pseudo-Gaucher cells [16].

The regular monitoring of GD patients for hematological malignancies is widely recommended [17]. A different, more difficult situation occurs when the patient first receives a diagnosis of an oncological hematological disease, and only as a result of in-depth diagnostics is a congenital metabolic disease diagnosed. We present a literature review inspired by the case of a Caucasian patient initially diagnosed with MM and later confirmed with a comorbid GD type 1 (GD1). We would like to point out the problem of delay and underdiagnosis in patients with Gaucher disease, especially in the population of patients with hematological diseases.

## 2. Case Report

A Caucasian 49-year-old man, not yet treated for any chronic diseases, reported periodically receding painfulness in the left hip for 3 months. Abnormalities observed in laboratory tests from the previous 5 years indicated persistent thrombocytopenia (98–100 10^3^/μL) with a slightly decreased erythrocyte count (RBC 3.22 10^6^/μL) and hemoglobin concentration (HGB 9.1 g/dL), along with mild yet stable enlargement of the spleen (14 cm in the ultrasound examination). During the following 2 months, chronic leg pain occurred, and the imaging tests followed by orthopedic consultation showed no significant pathological changes.

**Figure 1:**
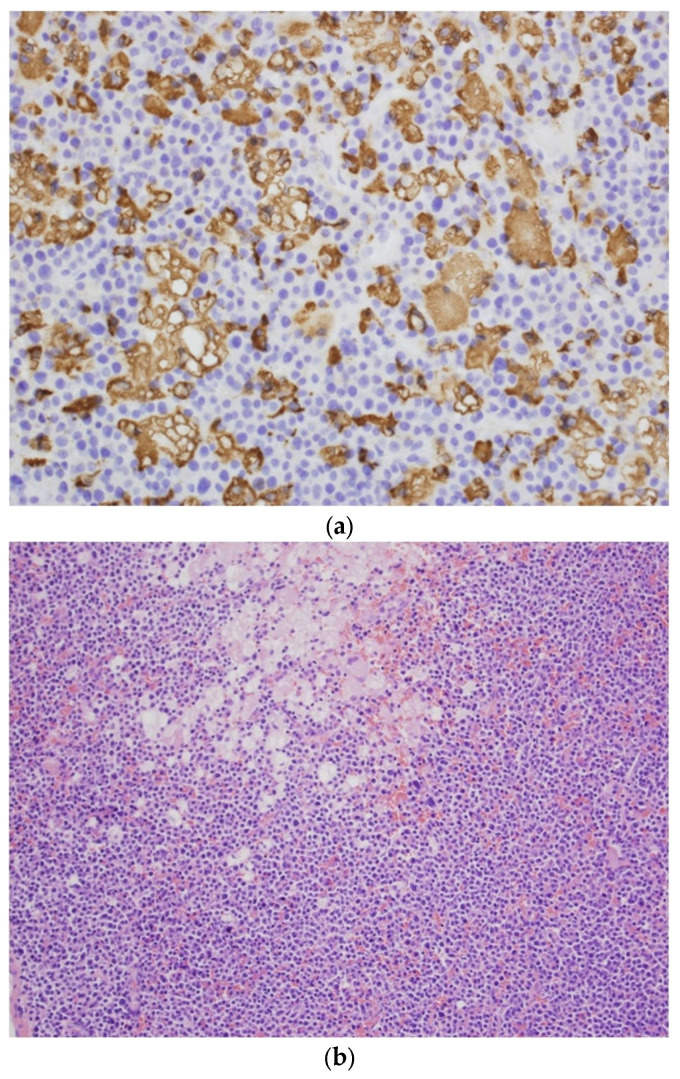
(**a**) CD68-positive abnormal histiocytes in the neoplastic plasma cell infiltration of the femoral bone. CD68 immunostaining. Obj. magn. ×40. (**b**) Neoplastic plasma cell infiltration of the femoral bone, with a focal accumulation of histiocytes with an abundant eosinophilic or clear cytoplasm (Gaucher’s cells). H&E. Obj. magn. ×20. The result of the re-analysis of the bone fragment collected by the hematopathologist from the pathological fracture.

Four months later, a pathological fracture of the left femoral shaft was observed, and osteosynthesis was performed to secure the fracture. At the same time, histopathological examination of the material taken from the fracture region confirmed extensive tumor plasmacytic infiltration, consisting of plasmocytes with moderate atypia, CD138+ /CD56+ /kappa- /lambda+, locally with the small clusters of histiocytes and with an abundant, weakly eosinophilic cytoplasm (Figure 1a,b). A primary diagnosis of solitary bone plasmacytoma with a local accumulation of histiocytes was established.

**Figure 2:**
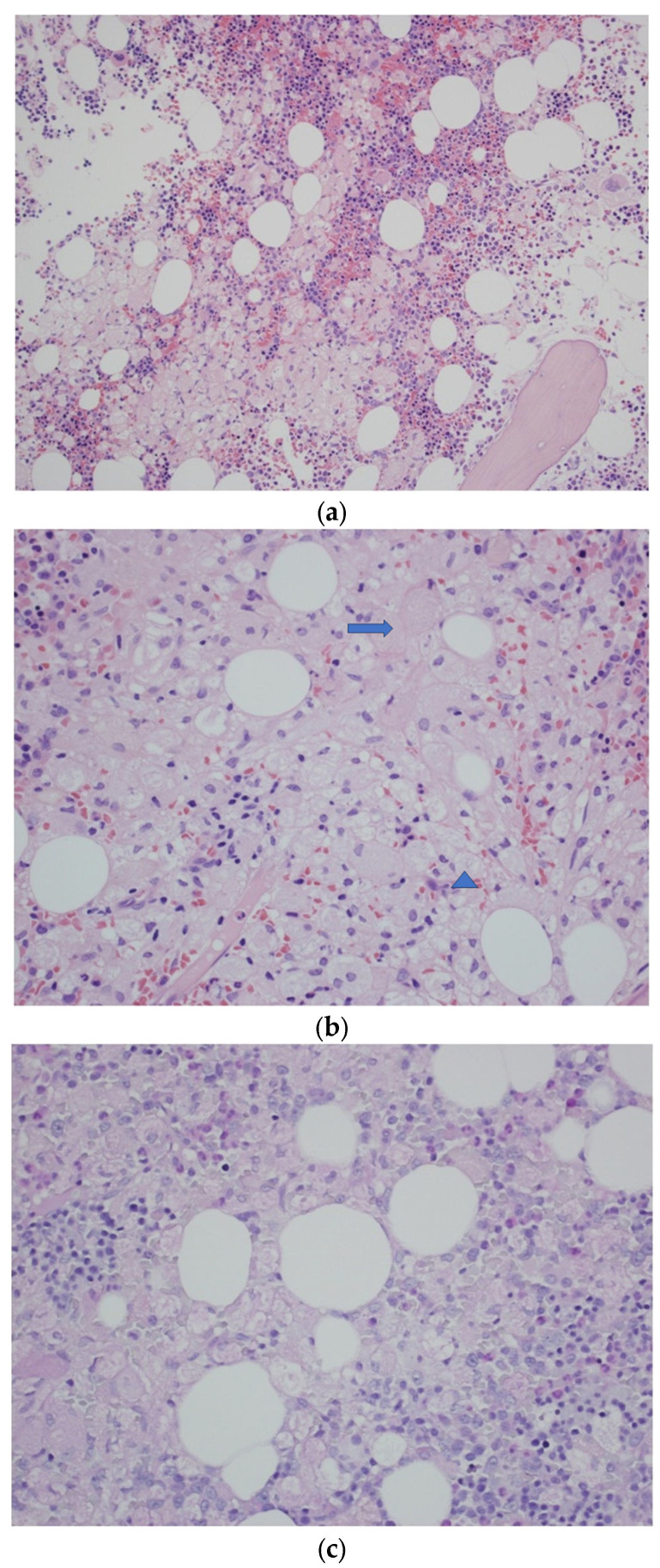

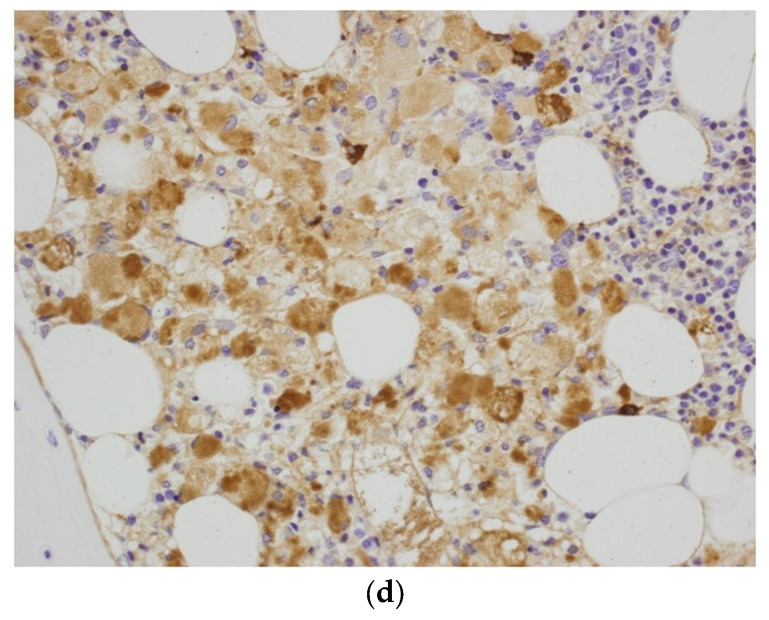
(**a**) Trephine biopsy—bone marrow occupied by the sheets of abnormal histiocytes. H&E. Obj. magn. ×20. (**b**) Trephine biopsy—a sheet of histiocytes (Gaucher’s cells) with an abundant eosinophilic (arrow) or clear cytoplasm with irregular vacuoles (arrowhead). H&E. Obj. magn. ×40. (**c**) Trephine biopsy—abnormal histiocytes with very weak cytoplasmic PAS positivity. Periodic acid-Schiff staining. Obj. magn. ×40. (**d**) Trephine biopsy—histiocytes stained for immunoglobulin kappa light chains. Kappa light chain immunostaining. Obj. magn. ×40.

In the Hematology Department, the laboratory tests revealed a decreased concentration of Hb > 9.3 g/L with elevated M protein levels of IgA 1.8 g/L, IgG 45.2 g/L, IgM 2.36 g/L, and serum calcium concentration 2.42 mmol/L, associated with the daily excretion of urinary monoclonal light chains (+), thus confirming the diagnosis of multiple myeloma. Chemotherapy with Melphalan, Prednisone, and Thalidomide was introduced. Three months after the diagnosis, despite the use of targeted treatment, a recurrent fracture of the left femur occurred above the previous anastomosis. The surgical resection of the changed fragment of the femur, with left hip joint endoprosthesis implantation, was performed. Despite continued high-dose chemotherapy, the patient’s hematological parameters did not significantly improve. Autologous stem cell transplantation (auto-HSCT) did not eliminate the problem of persistent thrombocytopenia, anemia, and immunity disorders. The control trephine biopsy revealed the dispersed plasmocytes CD138+, with a significant quantitative predominance of lambda cells (kappa/lambda approximately 1:6) constituting approximately 2–3% of the bone marrow cells. Approximately 80% of the bone marrow cells were CD68+ macrophages with an abundant, weakly PAS-positive, foamy or vacuolated, cytoplasm, without the obvious features of hemophagocytosis. A highly important morphological disorder in the histological picture was recognized: a scant population of neoplastic plasmacytes (constituting residual myeloma). Furthermore, due to persistent granulocytopenia (WBC 1.98 10^3^/μL) and thrombocytopenia (20 10^3^ /μL), a second auto-HSCT was performed. Again, this procedure did not satisfactorily improve patient’s condition. In the subsequent diagnostic trephine biopsy, a decreased number of megakaryocytes and Gaucher-like cells without visible hemophagocytosis were found in clusters, occupying approx. 70% of the biopsy area (Figure 2).

The reduced enzymatic activity of beta-glucosidase (32.57 μmol/L/h) in the peripheral blood leukocytes and elevated levels of chitotriosidase (1220 nmol/mL/h), as well as Lyso GL-1 (62.3 ng/mL), were detected. Genetic testing confirmed the homozygous GBA mutation p.N409S (also known as c.1226A>G and N370S; rs76763715); type 1 Gaucher disease was diagnosed. The combined clinical picture, confirmed by laboratory and genetic tests, made it possible to conclude a definitive diagnosis of comorbidity of MM and GD1 in this patient. The patient remained under the constant care of hematologists, having received maintenance chemotherapy and, in the meantime, he was admitted for enzymatic replacement therapy (ERT) using imiglucerase (Cerezyme) at an initial dose of 30 U/kg b.w., which was then increased to 45 U/kg body weight via intra-venal infusions every 2 weeks. The hematological parameters improved (PLT 60 10^3^ /μL, WBC 3.8 10^3^/μL, RBC 4.52 10^6^/μL), while the chitotriosidase level decreased (520 nmol/mL/h). The patient’s condition stabilized, and a good response to ERT was observed from the reduction in the spleen dimensions, stabilization of laboratory test results, and improvement of the patient’s quality of life. It was also concluded that there was no need to further increase the dose of the enzyme drug. After 8 months of improvement in the patient’s condition, there was a sudden progression of neoplastic changes. The patient ultimately died of acute respiratory failure with secondary cardiac arrest.

## 3. Discussion

This is the first case of a patient with the comorbidity of MM and GD1 described in Poland. This work draws attention to several important clinical issues. It focuses on the coexistence of two chronic and potentially fatal diseases, one hematological and one metabolic, in one patient. The increased predisposition to cancer development in Gaucher patients is well-known [12,13,18,19]. The article emphasizes the delay in the diagnosis of GD, as well as the overlapping of the effects of two diseases, which mutually change the clinical picture and prognosis for the patient.

Until recently, reports on cancers in GD were single-center reports. Previous publications have estimated the risk of multiple myeloma in GD to be 5.9 to 51.1 times higher, compared to the general population [19]. The latest publication by Rosenbloom et al., based on the data from the International Cooperative Gaucher Group registry, provides information on the relative risk of hematological and solid organ cancers and the association between gammopathies and MM in the GD1 population. This analysis was based on a large, multicenter, international population with a sufficient follow-up time for the development of malignancies. According to these data, the risk of MM was approximately nine times higher in the GD1 population in comparison with the general population of the United States [20]. In addition to hematological malignancies, the GD1 population had a higher risk of liver (2.9-fold), kidney (2.8-fold), melanoma (2.5-fold), and breast (1.4-fold) cancers [20].

The pathomechanism of this phenomenon, however, is not completely understood. It was shown that tumor-associated macrophages can promote tumorigenesis in non-GD patients [21,22]. Reports suggest that Gaucher cells are phenotypically related to tumor-associated macrophages, attract the migration of other macrophages, and promote differentiation towards tumor-associated macrophages [23,24,25]. Parallel GD is a disorder of numerical and functional macrophage abnormalities that includes disturbances in invariant NK-T cells that depend on sphingolipid metabolism to deliver a physiological response. Attention is drawn to the imbalance between pro-/antiproliferative sphingolipids in tumor cells. Ceramide (glucocerebroside) levels affect the length of cell survival: high levels stimulate apoptosis, autophagy, and cell cycle arrest, while low levels promote cancer cell survival [25,26]. Glucosylceramide deposition, chronic antigenic stimulation, signal transduction disorders such as PD1 expression, the cytotoxicity of T lymphocytes, increased free radical production, impaired antigen presentation, reduced intracellular ceramide levels, and disturbed autophagy have all been postulated to facilitate the growth of malignant clones in GD [22,25,27,28]. What is also worth noting is the importance of glucocerebrosidase (Gcase) in the process of autophagic cell death (as a positive mediator). The impairment of this process has the effect of inducing and sustaining the inflammatory process through constant inflammasome activation in human Gaucher macrophages [28,29]. Other hypotheses that might explain the relationship between Gaucher disease and lymphoproliferative disorders include disorders in intercellular transmission, e.g., the occurrence of a cytokine storm secretion of pro- and anti-flooded factors, cytokines/chemokines/hydrolases, or the influence of IL1/IL6, secreted by B lymphocytes, on the production of immunoglobulins [30,31,32]. Genetic factors in the process of carcinogenesis in patients with the GD1 population (genetic mutation, tumor phenotype) cannot be underestimated [24].

According to Watek et al., the potential pro-neoplastic mechanism of Gaucher cells and expression of programmed cell death protein 1 (PD-1) in tumor-associated macrophages is as follows: the accumulation of glucocerebroside in macrophages and expression of PD-1 on their surface affects the survival of tumor cells, and the cytokines they secrete increase their invasiveness and promote angiogenesis and the ability to form metastases. The prevalence of signals stimulating the survival of the neoplasm cell is also associated with the enzymatic blockade of ceramide formation; the result is failure to recognize the signal for cell death [25]. The order of diagnosis in the presented patient was disrupted; the genetically determined disease was diagnosed second. However, this demonstrates a significant delay in diagnosis and the problem of underdiagnosis in patients with GD [33,34]. Nonetheless, diagnostic delays are a significant problem for many patients, some of whom will have already experienced severe or irreversible late complications, unnecessary invasive diagnostic procedures, and even unnecessary/harmful treatments, like splenectomy, before the diagnosis of GD. In a survey of 212 patients with GD1 in the United States that aimed to examine the pre-diagnosis period, nearly one in six patients said they had not been diagnosed with an underlying condition over 7 years or more after their first consultation with a physician. In addition, many of them reported symptoms mostly associated with hematological disorders [35]. Mistry et al. based his work on surveys of 136 ERT-naive GD1 patients from the USA, Australia, and New Zealand and showed that in this group of patients, the time period from the first symptoms of GD to diagnosis was, on average, 49 ± 124 months in the USA and 36 ± 73 months in Australia/New Zealand. It is disturbing to note that at that time, for this reason, the average patient was assessed by an average of three specialists [36]. This is still a cause for concern and has unfortunately not improved significantly over the years; based on the description of a case with typical GD symptoms, only one in five hematologists considered GD in the first place [36].

The skeletal manifestations of GD and MM, such as vertebral fractures, osteoporosis, and bone marrow infiltration (focal/diffuse) on magnetic resonance imaging (MRI), are common features and require a differential diagnosis between the two conditions [37].

Another similarity between MM and GD is the association of these two diseases with polyclonal or monoclonal gammopathy, especially monoclonal gammopathy of undetermined significance (MGUS) [38]. It is widely regarded as a precancerous condition, with a risk of progression to multiple myeloma, especially when coexisting with serum-free light chains [39]. The 2022 registry showed higher age-adjusted incidence rates for MGUS [20]. This may prove the chronic lymphoproliferation of B lymphocytes and indirectly confirms the role of sphingolipids in stimulating their proliferation and the production of anti-lipid antibodies [40,41,42]. The role of saposin C as an antigen stimulating the production of antibodies in MGUS/MM is also emphasized [43]. The stimulation of cytokine release has also been suggested as a common mechanism of pathogenesis; however, the currently collected data are insufficient to link cytokine production in the bone marrow to MGUS in patients with Gaucher disease [17]. According to the World Health Organization, the age-standardized rate of incidence of multiple myeloma across the world in 2020 was 1.8 per 100,000 people [44]. The average age of onset of MM at diagnosis is 60–65 years, with only around 2% of patients being under 40 years of age. Previous risk studies of GD1 patients showed that patients with multiple myeloma were >50 years of age [6,19,45,46]. According to a US registry published in 2022, the age-adjusted risk of hematological malignancy was more than four times higher in GD1 patients, the risk of MM was approximately nine times higher, and the risk of NHL was approximately three times higher.

In contrast, the age-adjusted rate of MGUS in this study group was 445 per 100,000 persons for patients ≥18 years of age. The age-specific incidence rates of MGUS increased with age, especially at an age ≥50 years. The cumulative incidence of MGUS at age 60 was 6.6%. The time from GD1 diagnosis to treatment initiation did not affect the incidence of MGUS. The cumulative 10-year incidence of multiple myeloma among 126 patients with MGUS (n = 127) was 7.9%. The median time from the diagnosis of MGUS to MM was 3.5 (4.26) years. The African American population were separated from the above group due to their higher MGUS and MM index [20].

It was found that MGUS prevalence rates were unexpectedly high among younger patients, and the age of MM incidence was lower than previously estimated. On the other hand, the incidence of MM 10 years after the diagnosis of MGUS is similar to that in the general population (7.9% vs. 10%) [20]. Therefore, in the younger group, hematological metabolic vigilance is essential.

In addition to the sparse presentation of inherited metabolic disease (IMD) in the patient, another reason for the delayed diagnosis of IMD was the interpretation of the bone marrow biopsy result. Although the demonstration of enzyme activity deficiency, as well as genetic testing, is necessary for a definitive diagnosis of GD, bone marrow biopsy is still a common part of the diagnostic procedure for GD due to changes in the blood morphology pattern [2,47]. The presented clinical case underlines the diagnostic difficulties in Gaucher disease. Analyzing bone marrow biopsy material requires an experienced pathologist. The presence of Gaucher cells in the biopsy only confirms lysosomal storage disease in the patient and does not constitute grounds for the diagnosis of Gaucher disease. At the same time, the biopsy material may contain so-called pseudo-Gaucher cells, which are not related to LSDs [16]. Pseudo-Gaucher cells are identified in the bone marrow in various diseases, not only in MM but also in conditions such as chronic myelogenous leukemia, myelodysplasia, Hodgkin’s disease, thalassemia, or mycobacterial infection with atypical mycobacteria [48,49,50,51,52,53].

Gaucher cells do not differ in terms of immunohistochemistry from non-Gaucher reactive histiocytes (being CD68-positive with no aberrant expression of any antigen); thus, they can be identified only based on their morphology and, usually, PAS positivity. Classic Gaucher cells, as described in the literature, are plump histiocytes characterized by an abundant crumpled tissue-paper-like cytoplasm, usually with intensely positive periodic acid-Schiff staining (PAS) [8,54]. In the case described in our report, the morphological variants of Gaucher cells (very weak PAS staining, many cells with a clear and vacuolated cytoplasm) accumulated in high numbers (together with more typical cells), which was the reason for their misinterpretation during the first histopathological evaluation for reactive histiocytes accompanying neoplastic plasma cell infiltrates (resembling crystal storing histiocytes, though without crystalline material in the specimens examined). Additionally, the immunostaining of some histiocytes for immunoglobulin light chains and their accumulation in the bone fracture vicinity further suggested their reactive nature, secondary to plasma cell myeloma and its complications. An additional diagnostic difficulty is the fact that the cytomorphological picture of Gaucher cells is very diverse [55]. In the case described here, the role and type of histiocytes were not clear in the first histopathological examination, and this was one of the reasons why the primary histopathological diagnosis was focused on the process of hematological hyperplasia. Thus, the clinical picture and the course of therapy required further diagnosis of the patient, which led to the reassessment and the final, correct conclusion about the type of accumulated histiocytes. It is worth noting that the absence of plasma cells in the initial histological diagnosis has already been described in the literature, for example, in MM [56].

Already in the 1990s, bone marrow aspiration was not recommended as the primary diagnostic tool for GD [57]. Although the demonstration of Gaucher cells in a cytogenetic study often turnsthe diagnosis of Gaucher disease, it should be remembered that bone marrow biopsy is not the recommended procedure for this disease [2].

We demonstrated not only a very low content of Gaucher cells in the bone marrow biopsy but also the multiform manifestation of atypical Gaucher cells, whose frequency in bone marrow biopsies, according to the literature, ranges from 22 to 40% [57]. This is an important factor impeding the interpretation of the histopathological result and increasing the risk of false-negative results; still, a biopsy should be used to exclude the possibility of hematological disorders in the patient, rather than to diagnose GD [56,57].

Thus far, no official recommendations have been proposed for the treatment of patients with concomitant diseases, including GD and myeloma, even though the increased risk of co-occurrence of these diseases is emphasized [58]. Only a few reports are available in the literature showing an impaired response to targeted anti-cancer treatment in patients with GD [59,60,61,62]. Machaczka et al. claim that Gaucher cells increase the susceptibility of bone marrow cells to melphalan; this causes myelotoxicity with moderate effectiveness [62].

It seems, therefore, that failure in the treatment of a patient with MM, despite the complex and modern anti-cancer therapy described here, confirms the earlier clinical observations of a significantly worse response and the number of therapeutic complications in patients with cancer and GD [63]. It should also be emphasized that the demonstrated correlation between ERT doses and the clinical and laboratory effects of substitution therapy should be taken into account. Grabowski et al. demonstrated the best clinical response to targeted treatment among a group of patients with GD using an ERT dose >48 U/kg bw, compared to groups with lower doses. Taking into account the aggravating nature of comorbidities, such as neo-diseases, it seems that the pursuit of high, tolerated doses of ERT in these patients is a valid clinical assumption [64].

At the same time, our observation confirms that despite the positive impact of ERT on inherited metabolic disease, its effect on the progression from MGUS to active MM or MM treatment remains uncertain [13,38,65,66]. We can note that de Fost’s analysis suggests a beneficial effect of ERT on the occurrence and severity of gammopathy in patients with GD; out of the 63 analyzed patients, none of the 50 treated with ERT developed MGUS during ERT [61]. Research on this issue requires further observations, both clinical, as in this case report, confirming the positive effect of ERT on the course of MM in the early phase, as well as those shedding light on biochemical mechanisms in humanized animal models [67]. Based on our knowledge of the pathomechanism of this disease and its complications, treatment models are being developed. One of these is the advanced treatment of eliglustat, substrate reduction therapy (SRT). Oral therapy is offered to adult patients with GD of type 1 [68,69].

Currently, the potential use and effectiveness of this therapy in the treatment of complications/comorbidities of GD are being investigated. Laboratory reports have laid the groundwork for the use of SRT in patients with concomitant GD and hematological diseases. [41,42,70]. Some reports indicate a beneficial effect of SRT on paraproteinemia in GD in mouse models of the disease (with a decrease in antibodies against lyso-glucosylceramide (anti-LGL1), reduction in the level of clonal immunoglobulins, and reduction in malignant lymph proliferation) [41,70]. Nair et al., searching for the target antigen in GD-associated gammopathy, demonstrated a decrease in clonal immunoglobulin using eliglustat therapy in vivo in two GD patients with MGUS [42]. Further research on this issue, including clinical trials, is needed. At this point, however, there are insufficient data to determine the effect of ERT on the prevention of myeloma development as well as the modification of the response to treatment or the risk of recurrence of neoplastic disease. When monitoring patients with GD, we should maintain increased oncological vigilance. At the same time, attention should be paid to the potential co-occurrence of sphingolipidosis in patients undergoing hematological treatment.

## 4. Conclusions

It is necessary to annually monitor all adults with GD1 for the presence of immunoglobulins, free light chains, and M-spike. We would like to point out the underdiagnosis of GD and the problem of its late recognition. The diagnosis of MM in patients under 50 years of age should raise diagnostic doubts and stimulate the search for metabolic diseases like GD. The awareness of clinicians from various fields of medicine, especially hematologists and hematopathologists, about the relationship between storage and hematological diseases should be increased in order to perform diagnostics optimally and quickly and to avoid any unnecessary waste of time in establishing the final diagnosis. The presence of pseudo-Gaucher cells in the bone marrow may obscure the underlying pathology and contribute to misdiagnosis. Therefore, it is important that the biopsy material is analyzed by a pathologist experienced in the field of LSDs. A useful tool for increasing the number of GM diagnoses could be a registry of rare diseases, which is still too rarely recognized among clinicians.

An awareness of possible associations, appropriate immunohistochemistry, and appropriate additional testing based on clinical results is essential for a definitive diagnosis.

## Data Availability

Not applicable.
